# Sustainable Approaches for the Protection and Deprotection of Functional Groups

**DOI:** 10.1002/chem.202501387

**Published:** 2025-06-27

**Authors:** Ju Ha Jin, Mina An, Minki Jeon, Jaejeong Kim, Sung Min Kang, Isaac Choi

**Affiliations:** ^1^ Department of Chemistry Chungbuk National University Chungcheongbuk‐do 28644 Republic of Korea; ^2^ Daeso Plant, High Tech Pharm Chungcheongbuk‐do 27663 Republic of Korea

**Keywords:** electrochemistry, functional group (de)protection, photochemistry, redox chemistry, sustainable synthesis

## Abstract

While functional group tolerance is a critical consideration in synthetic method development, many functional groups often fail to remain intact under diverse reaction conditions. Therefore, the protection of specific functional groups and subsequent deprotection are convenient choices for the efficient and selective synthesis of target compounds, for which the strategies have been securely established. Meanwhile, the development of sustainable synthetic methods, particularly electrochemistry and photochemistry, has recently played a significant role in the field of organic synthesis, and these approaches have proven useful in the methods of functional group protection and deprotection. This review explores electrochemical and photochemical strategies for the protection and deprotection of functional groups, with a focus on their mechanistic diversity, sustainability, and synthetic utility across various functional group classes.

## Graphical abstract

Shield: Protection and deprotection are key to selective synthesis, but conventional methods can lack sustainability. This review highlights electrochemical and photochemical strategies as greener alternatives, emphasizing their mechanisms, functional group tolerance, and broad synthetic utility.

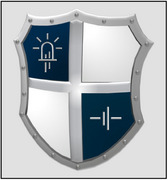

**Abbreviation**
bpy: 2,2′‐Bipyridine
Bz: Benzoyl
CSA: Camphorsulfonic acid
DIPEA: *N*,*N*‐Diisopropylethylamine
DMF: Dimethylformamide
IBX: 2‐Iodoxybenzoic acid
IPA: Isopropanol
TBA: Tetrabutylammonium
TBDPS: *tert*‐Butyldiphenylsilyl
TBHP: *tert*‐Butylhydroperoxide
TBS (TBDMS): *tert*‐Butyldimethylsilyl
TEA: Tetraethylammonium
TIPS: Triisopropylsilyl
TMA: Tetramethylammonium
TMS: Trimethylsilyl
MOP: 2‐Methoxy‐2‐propyl
Ms: Methanesulfonyl
NMP: *N*‐Methyl‐2‐pyrrolidone
PBS: Phosphate‐buffered saline
PVDF: Polyvinylidenefluoride

## 1 Introduction

### 1.1 Significance of Protection and Deprotection

**Figure 1:**
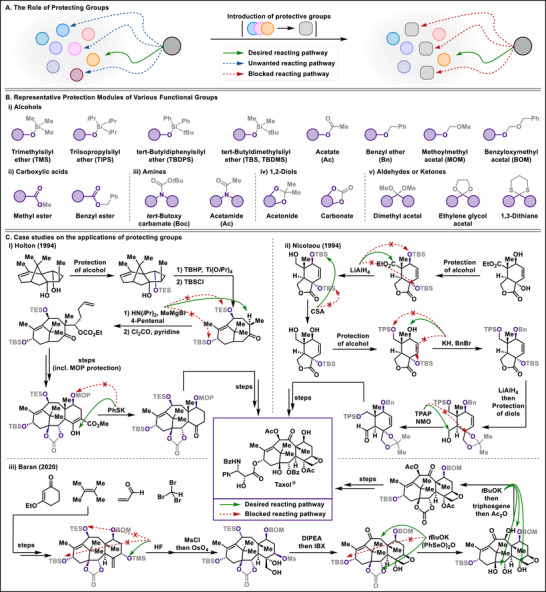
The role and types of protecting groups, and their application in the total synthesis of Taxol®.

Functional groups are a key structural unit of organic molecules that determines their chemical properties and reactivity. However, it is very difficult to selectively modify a single functional group, while discriminating against other reactive sites to suppress the formation of undesired byproducts. To tackle this common issue, the introduction of temporary derivatives, called protective groups, is the most useful and convenient approach, recognized as a well established technique among synthetic chemists.^[^ ^1^, ^2^, ^3^, ^4^, ^5^, ^6^, ^7^ ^]^ Consequently, one could achieve selective transformations among multiple reactive functional groups (Figure 1A).

While these procedures may appear straightforward, three critical considerations must be addressed when introducing a protective group:^[^ ^7^ ^]^ a) It is crucial to selectively protect only the desired functional group when introducing a protective group, thus often necessitating the use of mild reagents and conditions; b) It is imperative that the protective group remain stable and unaltered throughout the reaction; c) It is essential that, following the projected reaction, the protective group be easily removable without impacting other reactive sites. Since, no single protective group can meet all the necessary criteria and effectively protect functional groups in complex molecules, synthetic chemists are required to separately use different temporary protection methods for each specific requirement or application. Therefore, various protective groups have been developed and evolved according to their purposes and applications.

Arguably, one of the oldest protection methods for functional groups is acetal protection of various sugars with acetone, developed by Fischer in 1895,^[^ ^8^, ^9^ ^]^ under acidic conditions. Trityl ether protection, introduced by Baeyer in 1909,^[^ ^10^ ^]^ is another classical protecting strategy with a long history of application in organic synthesis. Following this, various examples of the diverse use of protective groups have been reported, but these processes are often associated with several limitations. Protection and deprotection reactions could be susceptible to unexpected side reactions—particularly when they do not meet the three requirements mentioned above—thereby complicating the prediction of synthetic outcome and potentially necessitating additional processes to remove side reactions. Therefore, synthetic chemists have come to recognize the need not only for developing new protective groups, but also for developing new approaches to protection and deprotection in order to synthesize more complex target molecules in an efficient fashion. For instance, in the progress of protective group development, the benzyl carbamate (Cbz) group was initially employed as a protective group for the *N*‐terminus of amino acids.^[^ ^11^ ^]^ It was revealed, however, that the long exposure under hydrogenolysis conditions used to remove this protective group is difficult to effectively apply to substances containing other labile functional groups. Therefore, alternative protective groups, such as *tert*‐butyloxycarbonyl (Boc)^[^ ^12^ ^]^ or 9‐fluorenylmethoxy carbonyl (Fmoc),^[^ ^13^ ^]^ were later developed and introduced as urethane‐based protection methods. Indeed, these approaches provided comparatively milder deprotection conditions, being acid‐labile or base‐labile, respectively.

Likewise, various protecting platforms have been extensively designed to enhance the performance and efficiency of projected reactions (Figure 1B). The majority of discovered protection methods focused on alcohols, which are notably labile under basic or oxidative conditions. These alcohols are typically protected as silyl ethers, alkyl ethers, or acetals. Additionally, diols can be protected by forming corresponding acetals with carbonyl groups or carbonates, while carbonyl groups, conversely, are protected as acetals or ketals. Acids and amines, frequently encountered in amino acid chemistry, are commonly protected as esters and amides, respectively. Despite this progress, finely tuned protecting groups have been further investigated to improve selectivity and removability during complex synthetic processes, ensuring that desired transformations occur without interference from reactive functional groups. Consequently, these strategies have been adopted across various fields of organic chemistry, demonstrating their effectiveness, particularly in total synthesis, which often involves multi‐step reactions and multiple reactive sites.

We have chosen Taxol, as a representative example of applying protecting groups in the synthesis of complex molecules. It is one of the most frequently attempted and reported molecules in the field of total synthesis research,^[^ ^14^, ^15^, ^16^, ^17^ ^]^ used as a chemotherapeutic agent against various types of cancer (Figure 1C).^[^ ^18^, ^19^ ^]^ Among the plethora of endeavors, Holton^[^ ^20^ ^]^ and Nicolaou^[^ ^21^ ^]^ independently reported an elegant synthesis of Taxol for the first time in 1994. A recent study from the Baran group, proposing a two‐phase synthesis, could also contribute strategically concise methods to the synthesis of this complex molecule.^[^ ^22^ ^]^ In Holton's approach, alcohols in the first two steps were protected by a silyl group, which helps avoid quenching of bases that are needed to deprotonate the alpha position of ketones for subsequent alkylation. Nicolaou also demonstrated a series of discerning choices for the protection of alcohols in their synthetic steps. The introduction of a silyl group in the first step prevented the deprotonation of alcohols while achieving the targeted reduction. Additionally, the orthogonal removal of the silyl group followed by protection with a benzyl group set the stage for a selective reduction process. Furthermore, the protection of the formed diol remained intact during oxidation, while the unprotected alcohol was transformed into an aldehyde. In Baran's synthetic method, which utilizes the previously studied Taxadiene,^[^ ^23^, ^24^ ^]^ the process of protection is minimized, and selective deprotection is employed to incrementally increase the level of oxidation in a precisely controlled manner.

### 1.2 Alternative Methods for Protection and Deprotection

The redox approach, distinct from classical reactions, has emerged as an alternative method for the protection and deprotection of functional groups. Notably, electrochemical and photochemical methods serve as prime examples of these innovations.^[^ ^25^, ^26^, ^27^, ^28^ ^]^ Over time, existing classical (de)protection methods have naturally evolved to favor milder and more user‐friendly conditions. As a result, significant advancements in synthesis using electrochemical and photochemical methods have yet to be widely demonstrated. However, electrochemical and photochemical approaches still offer notable advantages in terms of atom economy and redox efficiency. Traditional protection and deprotection strategies often require stoichiometric bases and introduce leaving groups, thereby diminishing atom economy and increasing chemical waste. Furthermore, conventional reductive deprotection frequently relies on strong reducing agents, which can compromise functional group tolerance. In contrast, redox‐driven methods provide milder conditions and improved selectivity, supporting more sustainable and functionally compatible transformations. For example, proton‐coupled electron transfer (PCET)^[^ ^29^, ^30^ ^]^ during electrooxidation often results in formal deprotonation at the anode, and the reduction of the proton at the cathode generates hydrogen gas. This approach permits the use of milder bases or none at all, while also bypassing the requirement for traditional leaving groups in electrophiles. Additionally, the injection of electrons into protected compounds via electro‐ or photoreduction avoids the use of harsh chemical reagents. This approach not only enhances functional group tolerance, but also reduces the generation of byproducts. Consequently, it enables milder reaction conditions and expands the range of substrates that can be efficiently deprotected without compromising the integrity of sensitive functional groups. From a sustainability perspective, these alternative methods for the installation and removal of temporary auxiliaries offer a promising avenue for transforming conventional methods into more sustainable and environmentally friendly alternatives.

### 1.3 Brief Introduction of Electron/Photon‐Driven Organic Synthesis

**Figure 2:**
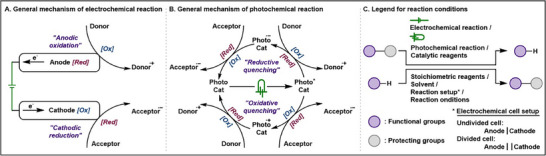
Electrochemical and photochemical strategies for functional group protection and deprotection.

Electrochemical organic synthesis is a field of study that explores the use of externally induced electron transfer to drive chemical transformations, offering a sustainable and selective approach to organic reactions (Figure 2A).^[^ ^31^, ^32^, ^33^, ^34^, ^35^, ^36^, ^37^, ^38^, ^39^, ^40^, ^41^, ^42^ ^]^ This process harnesses electrical energy to induce electrochemical reduction or oxidation, generating reactive intermediates that ultimately lead to the formation of target products. These transformations take place within electrochemical cells, where various factors, such as applied current, electrode materials, electrolyte composition, and cell configuration, play crucial roles in determining reaction efficiency, selectivity, and scalability.^[^ ^43^, ^44^, ^45^, ^46^, ^47^, ^48^ ^]^ By fine‐tuning these parameters, electrochemical synthesis enables precise control over reaction pathways, often eliminating the need for stoichiometric oxidants or reductants, thereby minimizing waste generation. This approach has gained increasing attention in modern synthetic chemistry due to its potential to enhance functional group tolerance, facilitate late‐stage functionalization, and contribute to the development of greener, more energy‐efficient methodologies.^[^ ^49^, ^50^, ^51^, ^52^, ^53^ ^]^ Despite these advantages, electrochemical organic synthesis often faces difficulties in scaling up due to uneven current distribution and the need for specialized equipment.^[^ ^54^, ^55^, ^56^, ^57^, ^58^, ^59^, ^60^, ^61^ ^]^

Photoredox catalysis is another field in which light energy triggers chemical transformations (Figure 2B).^[^ ^62^, ^63^, ^64^, ^65^, ^66^, ^67^, ^68^, ^69^, ^70^, ^71^, ^72^, ^73^, ^74^, ^75^ ^]^ Specifically, photochemical reactions absorb light energy and convert it into chemical energy. These reactions primarily utilize photoactive substances that absorb light of specific wavelengths, leading to changes in the electronic states of molecules and facilitating chemical transformations. Many photocatalytic reactions minimize the generation of byproducts and enable the efficient synthesis of desired compounds. Additionally, photochemical reactions offer an economical and environmentally friendly approach by using light as an energy source, thereby reducing the need for additional reagents or external energy input. Like electrochemistry, photochemistry plays a significant role in advancing modern technology and industry. However, challenges such as energy loss during light‐to‐chemical energy conversion and the limited stability of photocatalysts can hinder the efficiency and scalability of these reactions.^[^ ^76^, ^77^, ^78^, ^79^, ^80^, ^81^, ^82^ ^]^

A key feature of both electrochemical and photochemical methods is their ability to generate highly reactive intermediates under mild reaction conditions,^[^ ^83^ ^]^ often bypassing the need for harsh chemical reagents. Among these intermediates, radicals play a particularly important role, serving as fundamental driving forces in many electrochemical and photochemical transformations.^[^ ^84^, ^85^ ^]^ In electrochemical processes, electron transfer induces radical formation at controlled redox potentials, with reactivity fine‐tuned by electrode materials and current densities. Similarly, photoredox catalysis harnesses light energy to generate radicals. By adjusting oxidation‐reduction potentials, photosensitizers, and light wavelengths, both methods provide tunable control over radical pathways, making them valuable tools for functional group protection and deprotection.

While electrochemical and photochemical methods have garnered significant attention for their sustainability and efficiency in organic synthesis, translating these techniques from laboratory‐scale experiments to industrial applications remains a complex challenge. Although, they offer advantages such as mild reaction conditions, reduced reagent waste, and high functional group tolerance, their scalability is often hindered by efficiency losses, catalyst stability issues, and material durability concerns. In addition to scalability of both reaction methods, factors such as the need for specialized equipment, precise reaction control, and cost‐effective implementation further complicate their widespread adoption. Addressing these challenges requires continuous advancements in catalyst design, reaction optimization, and scalable process development. By overcoming these hurdles, electrochemical and photochemical methods have the potential to become integral to large‐scale industrial synthesis,^[^ ^86^ ^]^ paving the way for more sustainable chemical manufacturing.

### 1.4 The Aim and Scope of the Review

This review explores electrochemical and photochemical strategies for the protection and deprotection of functional groups, emphasizing their mechanistic proposal and synthetic utility. Unlike conventional methods that often rely on stoichiometric reagents, toxic chemicals, and harsh conditions, these emerging techniques enable selective transformations under milder, more efficient, and sustainable conditions. As modern synthetic chemistry increasingly demands atom‐ and step‐economical processes with improved environmental compatibility, electrochemical and photochemical approaches offer valuable alternatives by enabling on‐demand reactivity via electrical input or light energy. The content of this review is organized by functional group class: alcohols, amines, carboxylic acids, amides, diols/amino alcohols, and others, highlighting key developments in each category. Special attention is given to reaction mechanisms, redox principles, and the nature of intermediates that govern (de)protection reactivity. Also, representative examples are provided to demonstrate their synthetic applicability. Although, photolabile protecting groups (PPGs) are partially addressed in this review, their overall scope is vast and has been thoroughly covered in other dedicated reviews.^[^ ^87^, ^88^, ^89^ ^]^ Therefore, in this work, the discussion of PPGs is intentionally limited and presented only in relation to specific functional group classes, in keeping with the organizational focus of the review.

All abbreviations for protecting groups have been provided, and reaction conditions have been preserved as closely as possible to the original text to ensure clarity and reproducibility. For other conventions referenced throughout the review, please see Figure 2C. The list of examples is not intended to be comprehensive, and any omissions are unintentional.

## 2 Alcohols

Alcohols are defined as organic compounds that contain one or more hydroxyl groups attached to carbon atoms.^[^ ^90^, ^91^ ^]^ They serve as ubiquitous and amphoteric building blocks in a plethora of chemical and biological processes and exist in various forms for a wide range of purposes. For example, methanol and ethanol are utilized in various applications, including industrial solvents, fuels, cosmetics, and food products. Consequently, protecting groups are often required to temporarily mask the reactivity of alcohols during complex synthetic sequences.

### 2.1 Protection

#### 2.1.1 Acetyl Protection

Among various approaches for the protection of alcohols, acetylation is one of the easiest methods among others. To achieve the acetylation of alcohols,^[^ ^92^ ^]^ acetic anhydride or acetyl chloride is typically employed to corresponding alcohols in the presence of pyridine analogs. Pyridine is a readily available Lewis base, playing a role in the neutralization of byproducts in this protection manifold. However, pyridine is associated with toxicity concerns and potential health risks, in addition to its strong odor.

**Scheme 1:**
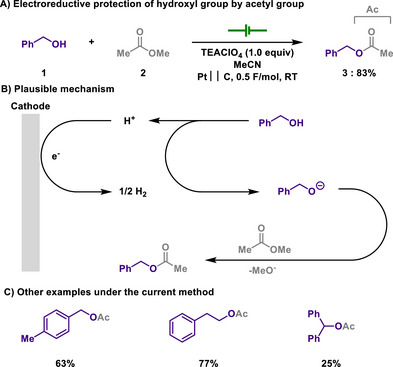
Electroreductive protection of hydroxyl group by acetyl group.

In this regard, Nakajima demonstrated electrochemical acetyl (Ac) protection of alcohols through transesterification strategy^[^ ^93^ ^]^ under mild conditions (Scheme 1).^[^ ^94^ ^]^ The reaction is initiated by the formation of an alkoxide anion, while hydrogen evolution reaction occurs as a cathodic reduction event. Although, a significant excess of methyl acetate was required as an acetylation source, the current efficiency was recorded between 126% and 166%, showing a high level of its energy efficiency. Therefore, the use of additional base could be avoided, with MeOH being the sole byproduct.

#### 2.1.2 MOM Protection

The significance of using the methoxymethyl group (MOM) for alcohol group protection lies in its convenience of installation and removal. The protection process generally involves deprotonation of targeted alcohols, followed by the addition of chloromethyl methyl ether (CMME), while acidic media leads to the deprotection of the MOM group. Despite the easiness of the reaction manifold, CMME is known to be a carcinogen and is heavily regulated due to its hazardous nature when used.

**Scheme 2:**
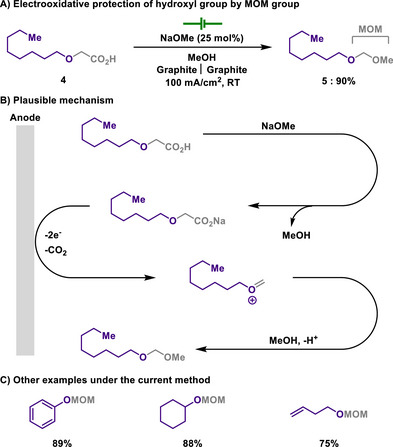
Electrooxidative protection of hydroxyl group by MOM group.

From this perspective, Lam investigated an environmentally friendly and safe approach for the MOM assembly (Scheme 2).^[^ ^95^ ^]^ The reaction proceeded in two steps: the synthesis of *α*‐alkoxy carboxylic acids from alcohols, followed by the subsequent formation of MOM ethers via electrochemical reactions. The *α*‐alkoxy carboxylic acids were chemically prepared through the treatment of alcohols with bromoacetic acid. Following this, electrochemical decarboxylation and a subsequent radical‐polar crossover process generate carbocations, which are quenched by methoxide, enabling MOM protection overall. The two‐step approach was not significantly affected by steric hindrance, making it compatible with various functional groups, and neither the current density nor the type of base influenced the reaction performance, providing a general and robust method for MOM protection.

#### 2.1.3 Benzyl Protection

The benzyl (Bn) group is also a commonly used moiety for protecting alcohols. The conventional method for introducing a benzyl group involves an S_N_2 reaction with benzyl halides, known as the Williamson etherification reaction,^[^ ^96^ ^]^ which requires bases such as potassium hydride or sodium hydride under moisture‐free conditions. However, this reaction often becomes inefficient for synthesizing ethers with sterically hindered structures. Additionally, the bases used are associated with halides that are combustible, pose reproductive toxicity, and are classified as Group B carcinogens.

**Scheme 3:**
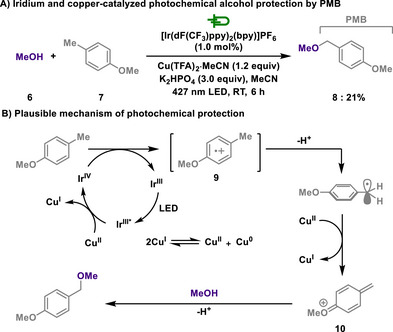
Iridium and copper‐catalyzed photochemical alcohol protection by PMB.

In this aspect, Yoon group showed benzyl protection of alcohols via photoredox catalysis, in which photoredox oxidation and radical‐polar crossover processes are orchestrated by iridium catalyst and copper salt, respectively (Scheme 3).^[^ ^97^ ^]^ The wise choice of copper oxidants leads to suppressing the generation of reactive oxygen intermediates, while the photocatalyst is selected to be sufficient for both the oxidation of arenes and the reduction of copper(II) salt. The iridium photocatalyst undergoes an oxidative quenching cycle, allowing arenes **7** to be oxidized to **9**. Interestingly, both the iridium(IV) species (E_1/2_ = +1.94 V) and the iridium(III)* species (E_1/2_ = +1.65 V) are capable of oxidizing the arene substrate (E_p/2_ = +1.52 V). The unstable radical cation undergoes deprotonation and subsequent oxidation by radicalophilic Cu(II) to form the quinone methide cation **10**, whereby the presence of alkoxy substituents on the arene is essential for stabilizing this intermediate. Lastly, nucleophilic attack of alcohol leads to the formation of the benzyl ether **8**. Although, primary benzylation is less effective under the reaction conditions due to the overoxidation, this reaction manifold was applicable to a broad range of alcohols, thus emphasizing a high functional group tolerance.

### 2.2 Deprotection

#### 2.2.1 Benzyl Deprotection

As discussed in Section 2.1.3., alcohols can be readily protected by benzyl groups. Benzyl ethers are particularly valued for their good stability, making them reliable protecting groups in various synthetic applications. In stark contrast to their ease of installation and robustness, their removal is typically less practical, often requiring harsh conditions such as transition metal‐catalyzed hydrogenation in the presence of hydrogen gas or Birch‐type reduction. Hydrogenation with molecular hydrogen poses significant risks due to its nature of explosiveness, while the catalysts employed in the reaction typically consist of economically demanding precious metals like palladium or platinum, deposited on a porous carbon support, more or less prone to spontaneous ignition.^[^ ^98^ ^]^ Moreover, the Birch reduction is notorious for its less user‐friendly reaction conditions, which typically involve the use of flammable alkali metals, such as sodium or lithium, under cryogenic temperatures. Therefore, these methods are less suitable when substrates contain easily reducible functional groups.

**Scheme 4:**
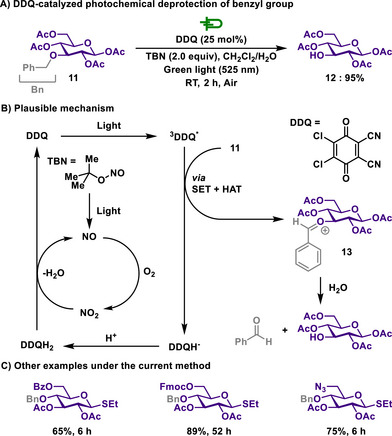
DDQ‐catalyzed photochemical deprotection of benzyl group.

However, Seeberger and Pieber proposed a milder photochemical approach for benzyl group deprotection, using 2,3‐dichloro‐5,6‐dicyano‐1,4‐benzoquinone (DDQ) as a catalyst (Scheme 4).^[^ ^99^ ^]^ In this method, *tert*‐butyl nitrite (TBN) is employed as a co‐catalyst, allowing DDQ to be used catalytically rather than stoichiometrically. Upon light irradiation, DDQ reaches its triplet excited state, initiating the formation of an oxocarbenium intermediate of benzyl‐protected carbohydrate **13** through the combination of single electron transfer (SET) and hydrogen atom transfer (HAT). Simultaneously, TBN releases nitric oxide, which subsequently forms nitrogen dioxide (NO_2_) as an oxidant, reoxidizing DDQH_2_ back to DDQ. Due to DDQ's weak absorption, reactions at wavelengths above 450 nm result in prolonged reaction times, potentially leading to overoxidation and product degradation. However, batch reactions at 525 nm exhibited superior suppression of byproduct formation compared to those at 440 nm, and continuous‐flow conditions further enhanced the efficiency of light irradiation while reducing reaction time without compromising selectivity.

**Scheme 5:**
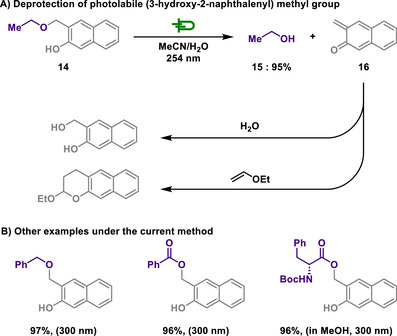
Deprotection of photolabile (3‐hydroxy‐2‐naphthalenyl) methyl group.

As an extension of benzyl group deprotection, Popik introduced 3‐hydroxy‐2‐naphthalenemethanol as a PPG not only for alcohols, but also for carboxylic acid and phenols, demonstrating efficient photolysis across a wide range of substrates with high functional group tolerance (Scheme 5).^[^ ^100^ ^]^ Notably, PPGs, also known as caged compounds, allow for precise spatial and temporal control over the release of biologically active molecules through light activation. First introduced by Barltrop in 1962,^[^ ^101^ ^]^ PPGs have since been chemically diversified to enable controlled release of a wide array of functional groups, and their utility has also been demonstrated in complex molecule synthesis.^[^ ^102^, ^103^ ^]^ In the Popik's work, phenols and naphthols exhibit enhanced acidity in the excited state, enabling excited‐state intramolecular proton transfer (ESIPT), typically forming short‐lived zwitterionic intermediates that rapidly revert to the starting material. However, in *o*‐hydroxybenzyl alcohol derivatives, ESIPT induces C─O bond cleavage in an intramolecular fashion, leading to the formation of *o*‐quinone methide instead of reverting. The reaction proceeds through the formation of 2‐naphthoquinone‐3‐methide (NQM), a highly reactive intermediate. Unlike typical *o*‐quinone methide species, which undergo rapid reversion, NQM is either deactivated in hydroxylic solvents or efficiently trapped as a photostable Diels‐Alder adduct with ethyl vinyl ether, further stabilizing the reaction product. Moreover, the PPG undergoes highly efficient photolysis, achieving a quantum yield of approximately 0.2 upon exposure to 254 or 300 nm irradiation, allowing for the selective release of substrates in good chemical yield. Additionally, this photolabile system exhibits rapid deprotection kinetics, with a half‐lifetime of substrate release below 10 µs, ensuring fast photochemical transformations.

#### 2.2.2 PMB Deprotection

**Scheme 6:**
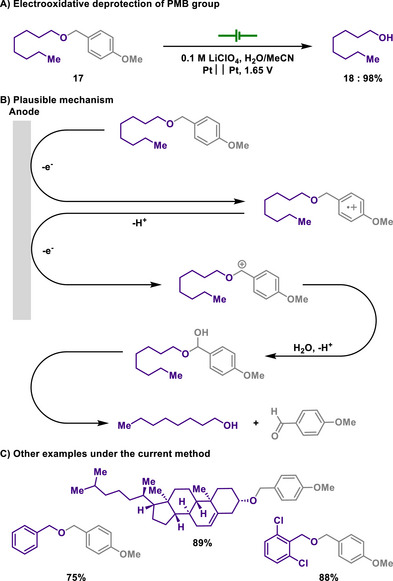
Electrooxidative deprotection of PMB group.

As an alternative method for the simple benzyl protective group, the *para*‐methoxybenzyl (PMB) group can also shield various alcohols and phenols. Unlike the deprotection with the benzyl group, the deprotection with the PMB group involves oxidative methods, with DDQ being a commonly used reagent.^[^ ^104^, ^105^ ^]^ However, it is important to note that DDQ reacts with water to produce highly toxic hydrogen cyanide. Regarding this, Weinreb and Epling reported an electrochemical method for the mild deprotection of PMB group using controlled potential electrolysis (Scheme 6).^[^ ^106^ ^]^ Since the benzyloxy group has sufficiently low oxidation potential, it renders benzyl protected alcohols amenable to being electrochemically oxidized. Therefore, two‐electron oxidation accompanied by deprotonation enables the formation of an oxocarbenium ion, which is subsequently hydrolyzed to afford corresponding alcohols and aldehydes. Remarkably, this deprotection process proceeds smoothly across a broad range of substrates, from simple alcohols to structurally complex molecules like cholesterol, owing to the use of controlled‐potential electrolysis.

**Scheme 7:**
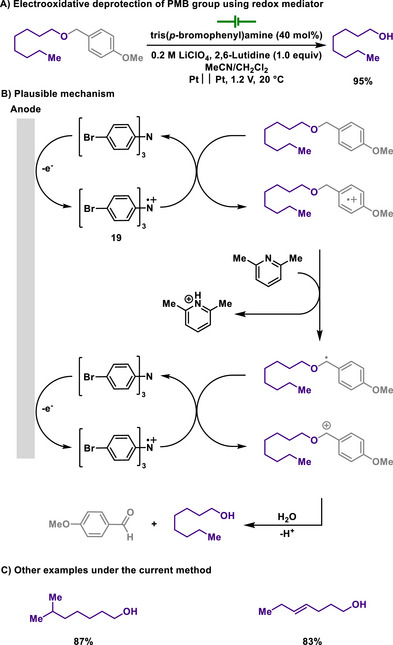
Electrooxidative deprotection of PMB group using redox mediator.

In contrast to Weinreb's work, which utilized controlled potential electrolysis for the deprotection of the PMB group, Steckhan introduced a milder method for the removal of the PMB group using tris(*p*‐bromophenyl)amine as a redox mediator (Scheme 7).^[^ ^107^ ^]^ The redox shuttle generates a stable cation radical under electrooxidation conditions, thus playing a role in mediating the electron transfer.^[^ ^108^ ^]^ The reaction is initiated by the electrooxidative generation of amine radical cation **19**, which indirectly oxidizes PMB‐protected alcohols. Only catalytic amounts of triarylamine are utilized as an electron transfer mediator, leading to the reaction proceeding with excellent yields. Under the reaction conditions, double bonds remain intact, while the very low oxidation potential of the redox mediator allows for broader functional group tolerance. However, the author reported that direct anodic deprotection requires an anode potential of at least +1.6 V versus SCE and is often accompanied by electrode fouling issues.

**Scheme 8:**
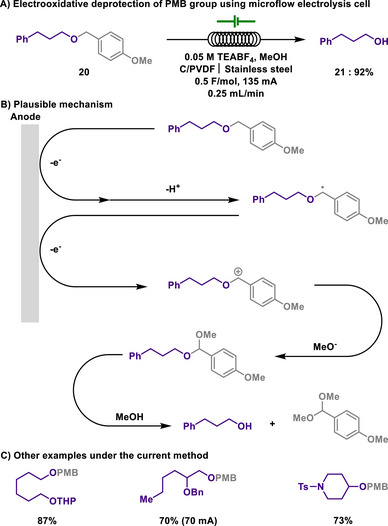
Electrooxidative deprotection of PMB group using microflow electrolysis cell.

For industrial applications of PMB deprotection, Brown and co‐workers developed a scalable and sustainable electrochemical method using a microflow electrolysis cell, achieving efficient cleavage without the need for stoichiometric chemical oxidants (Scheme 8).^[^ ^109^ ^]^ The reaction proceeds in methanol with tetraethylammonium tetrafluoroborate as a recyclable electrolyte, and generates *p*‐methoxybenzaldehyde dimethyl acetal as a benign byproduct. The system was successfully applied to a wide range of PMB‐protected substrates, including aliphatic or aromatic alcohols. Selective deprotection was observed even in the presence of other commonly used protecting groups, such as TBDPS, tetrahydropyranyl ethers (THP), and benzyl groups. The continuous‐flow design enabled facile scale‐up, achieving productivities up to 7.5 g/hour and isolating up to 63 g of deprotected alcohol in a single run, setting the stage for the practicality of this method. Therefore, this method outperformed conventional DDQ‐ or CAN‐based protocols in terms of sustainability.

**Scheme 9:**
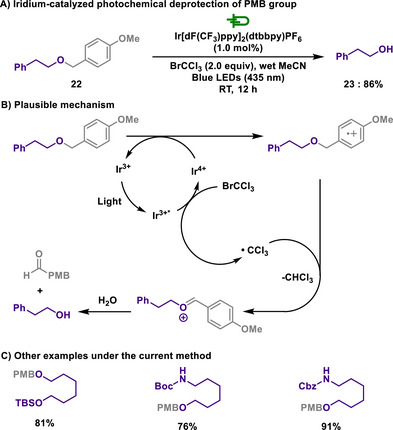
Iridium‐catalyzed photochemical deprotection of PMB group.

The deprotection of PMB groups is not confined to electrochemical techniques but extends to the realm of photoredox catalysis. The Stephenson group developed a visible‐light‐induced PMB deprotection employing an iridium‐based photocatalyst in the presence of tetrahalomethane as an oxidative quencher (Scheme 9).^[^ ^110^ ^]^ Upon excitation, the iridium photocatalyst enters its oxidized state, facilitating the generation of a trichloromethyl radical from BrCCl_3_. The oxidized iridium species subsequently oxidizes the PMB‐protected alcohol, which undergoes hydrogen abstraction by the trichloromethyl radical followed by hydrolysis, yielding the corresponding alcohol and *p*‐anisaldehyde. This method enabled the highly selective removal of PMB groups while fully tolerating other protective groups, such as TBS, Boc, or benzyloxycarbonyl (Cbz) groups.

**Scheme 10:**
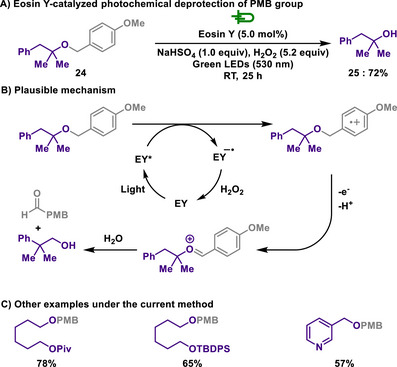
EY‐catalyzed photochemical deprotection of PMB group.

Similarly, the Cheng group reported the deprotection of PMB groups using Eosin Y (EY) as a photocatalyst together with hydrogen peroxide as the terminal oxidant under acidic conditions (Scheme 10).^[^ ^111^ ^]^ In comparison to Stephenson's pioneering work, this method utilized longer‐wavelength light, providing a milder reaction protocol while maintaining similar levels of chemoselectivity. To evaluate the scalability of the photoredox catalytic protocol, a scale‐up experiment was conducted by increasing the total LED output to 24 W while maintaining the standard reaction setup, yielding comparable results.

**Scheme 11:**
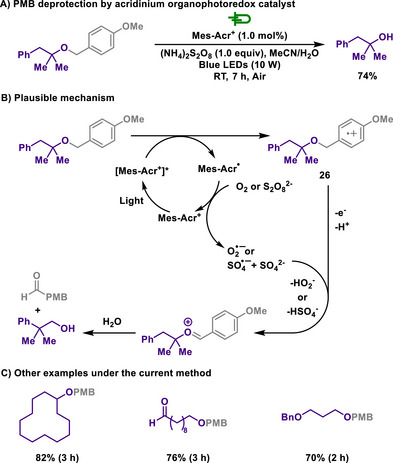
Acridinium‐catalyzed photoreductive deprotection of PMB group.

Furthermore, the Woo group demonstrated a photoredox deprotection of PMB groups employing an acridinium‐based photocatalyst (Scheme 11).^[^ ^112^ ^]^ These approaches underscore an environmentally friendly strategy by utilizing a metal‐free photoredox catalyst, further broadening the scope of sustainable PMB deprotection methodologies. In the presence of an excited photocatalyst, PMB ether undergoes oxidation to form the radical cation **26** via SET, simultaneously generating a reduced photocatalyst. External oxidants act as co‐oxidants, oxidizing the reduced catalyst to complete the photocatalytic cycle. Furthermore, when these two reagents are used together, the catalytic activity of the cycle increases, accelerating the formation of the oxocarbenium ion. Oxocarbenium cations generated by superoxide radical anions or sulfate radical anions undergo hydrolysis to form hemiacetal intermediates, which subsequently yield deprotected alcohols. Similar to previous studies on electrochemical and photochemical PMB removal, this reaction method also presented selective deprotection of the PMB group from ethers containing protecting groups such as acetyl, benzyl, and pivaloyl (Piv). Ethers bearing acid‐sensitive protecting groups such as TBS, TBDPS, and THP exhibited a moderate decrease in product yields. This was attributed to the acidification of the reaction solution due to the formation of bisulfate anions during the reaction. Furthermore, increasing the intensity of illumination to 40 W in gram‐scale experiments provided a comparable outcome for the deprotected alcohol.

#### 2.2.3 Benzoyl Deprotection

Benzoyl groups are frequently used for the protection of alcohols, which affords benzoates.^[^ ^113^, ^114^ ^]^ Benzoyl chloride or benzoic acid are typically used for protection, while the deprotection of benzoyl groups requires acids or bases, generally necessitating subsequent neutralization. Although this deprotection manifold provides a simple and efficient recovery of the target alcohols, electrochemical approaches have been shown to eliminate the need for bases or acids.

**Scheme 12:**
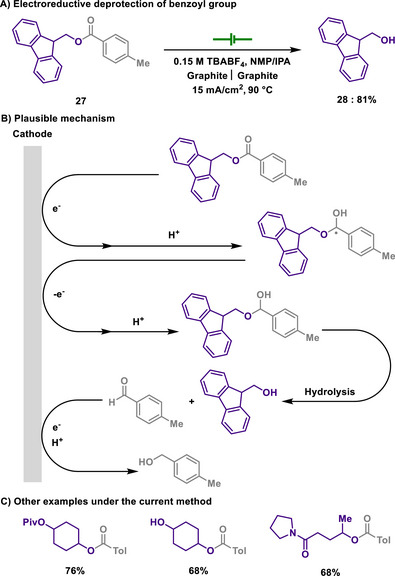
Electroreductive deprotection of benzoyl group.

In 2009, Marko discovered the electrochemical method for the removal of benzoyl group, thus allowing alcohols to be restored (Scheme 12).^[^ ^115^ ^]^ While electrochemical reduction of esters provided a radical anion that is prone to be fragmented to acetate in aprotic reaction medium, they proposed that the addition of a proton source, isopropanol, curbed the reversibility of the reduction process, and thus the undesired pathway of decomposition was suppressed. Notably, the developed deprotection method exhibits excellent compatibility with various functional groups and protecting groups, including aliphatic esters, amides, alcohols, silyl ethers, acetals, and ketals. In addition to this, dialing the reaction potential applied allows for the selective deprotection of differently substituted benzoyl groups. Also, this electrochemical approach showed a comparable performance to SmI_2_‐mediated conditions, offering a valuable and sustainable tool for the removal of benzoyl protectives.

#### 2.2.4 Carbonates Deprotection

Carbonates are also widely used as protecting groups for hydroxyl groups. Generally, carbonate protecting groups are readily hydrolyzed under basic conditions; however, their reactivity can vary significantly depending on the type of alkyl substituent of the carbonates. Representatively, 2,2,2‐trichloroethyl carbonate (Troc) protection can be reductively removed by using zinc reductant in acidic media. Although these carbonate groups are readily accessible protection methods in synthesis, they also present several challenges. For instance, excessive zinc exposure poses environmental risks, particularly to aquatic organisms, and can be toxic to the human nervous system.

On the other hand, the isonicotinyloxycarbonyl (*i*Noc) group, first developed by Veber,^[^ ^116^ ^]^ is another protecting group, designed for the amino group of lysine residues. In Veber's seminal study, the *i*Noc group was removed using a Zn(II) reductant or via electroreduction with a mercury cathode.

**Scheme 13:**
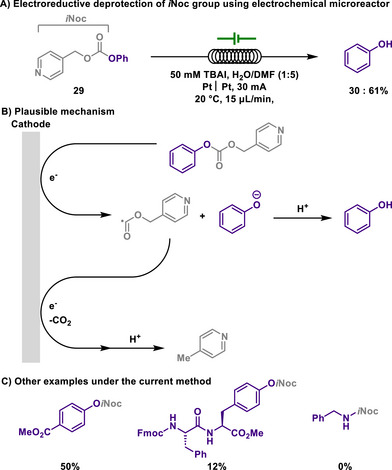
Electroreductive deprotection of *i*Noc group using electrochemical microreactor.

Wirth developed a more efficient method for the removal of *i*Noc‐protected alcohols using an electrochemical microreactor, thereby enabling the recovery of the alcohols. (Scheme 13).^[^ ^117^ ^]^ Indeed, the proximity of the platinum electrodes in the continuous‐flow reactor offers a significant advantage, allowing efficient electrochemical redox reactions and rapid deprotection with minimal electrolyte concentration. In contrast, batch conditions exhibited lower reaction performance despite extended reaction times, highlighting the superior efficiency of the electrochemical microreactor. The electrochemical deprotection of the *i*Noc group offers not only operational advantages, but also broad substrate applicability, including compatibility with tyrosine‐containing peptides. Additionally, the deprotection manifold was highly selective, effectively discriminating against *i*Noc‐protected amines, which remained intact under the identical electrochemical conditions. In other words, it was revealed that the *N*‐*i*Noc group (carbamate) remains highly stable under the applied electrolysis conditions, while *O*‐*i*Noc (carbonates) and *S*‐*i*Noc (thiocarbonates) undergo facile deprotection. This is attributed to the more positive reduction potentials of *O*‐*i*Noc and *S*‐*i*Noc compounds compared to *N*‐*i*Noc under the reaction conditions.

**Scheme 14:**
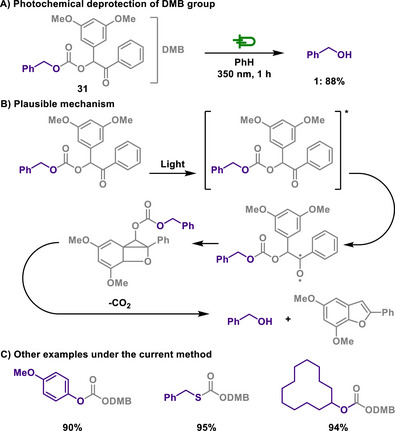
Photochemical deprotection of DMB group.

Photochemical strategies have also been explored as an alternative approach for selective deprotection of carbonate‐based protecting groups. In this context, Pirrung introduced a new class of protecting group, 3,5‐dimethoxybenzoin (DMB),^[^ ^118^ ^]^ that can be photochemically removed from protected alcohols (Scheme 14).^[^ ^119^ ^]^ These DMB carbonate groups can be readily removed within a short period of time. It is noteworthy that the concomitant formation of the benzofuran derivative offers significant advantages for assessing the degree of deprotection due to its strong fluorescence.

**Scheme 15:**
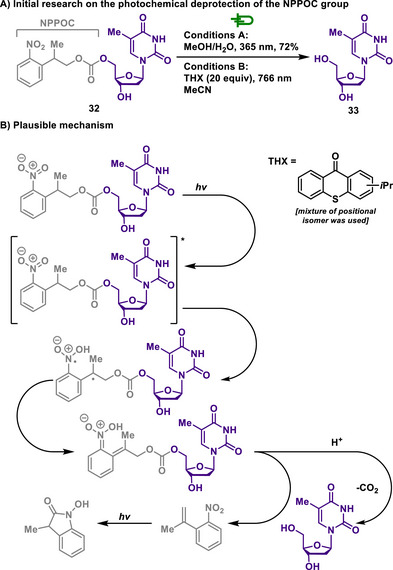
Photochemical deprotection of the NPPOC group.

In 1998, Pfleiderer first reported the synthesis and characterization of the nitrophenylpropyloxycarbonyl (NPPOC) group as one of the PPGs for nucleosides and nucleotides.^[^ ^120^ ^]^ Upon UV irradiation, the NPPOC group undergoes a photoinduced *β*‐elimination. This leads to rapid cleavage and formation of a styrene derivative as a key intermediate. The intermediate is photochemically unstable and further converts into a stable product like *N*‐hydroxyoxindole (Conditions A, Scheme 15). While the conventional NPPOC group exhibits high efficiency in one‐photon photochemical deprotection, it demonstrates low sensitivity to two‐photon excitation (TPE), making it challenging to achieve selective deprotection at a desired spatial location. However, by employing thioxanthone (THX) as a sensitizer, the two‐photon absorption efficiency of NPPOC could be significantly enhanced without requiring modifications to the NPPOC structure itself, developed by Pirrung (Conditions B, Scheme 15).^[^ ^121^ ^]^ To explore this approach, one‐photon‐sensitized deprotection mediated by THX was first investigated, followed by its extension to two‐photon‐assisted (TPA) deprotection. In the case of one‐photon deprotection using 366 nm, an increase in THX concentration resulted in a decrease in the reaction rate, which was attributed to the quenching of the THX triplet state by molecular oxygen. In contrast, in the case of two‐photon sensitization using 766 nm fs‐pulsed, the reaction rate increased with higher THX concentrations, reaching maximum efficiency at an optimal concentration of 20 equivalents.

These findings introduce a modular strategy for optimizing two‐photon deprotection by systematically varying the sensitizer while retaining well‐established protecting groups. This approach holds significant potential for applications ranging from biological effector release to direct‐write molecular photolithography.

#### 2.2.5 Silyl Deprotection

Silyl group is a versatile protecting moiety used to shield alcohols under basic conditions.^[^ ^122^ ^]^ It is noteworthy that as the size of silyl protecting group increases, the resistance against hydrolysis is typically enhanced.^[^ ^123^, ^124^ ^]^ Such protean silyl protecting group can be easily removed under acidic conditions or in the presence of fluoride sources, such as tetrabutylammonium fluoride.

**Scheme 16:**
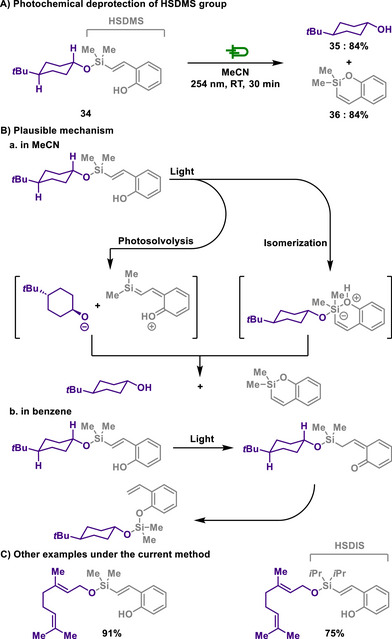
Photochemical deprotection of HSDMS group.

Pirrung and Lee developed two novel silyl protecting groups, (hydroxystyryl)dimethylsilyl (HSDMS) and (hydroxystyryl) diisopropylsilyl (HSDIS), which can be efficiently removed upon irradiation of UV light.^[^ ^125^ ^]^ The styrylsilyl groups were readily installed to primary and secondary alcohols, and deprotection occurs also within a short reaction time (Scheme 16). Mechanistic studies suggest that deprotection proceeds through photoinduced *trans‐cis* isomerization, enabling intramolecular elimination or rearrangement pathways. Additionally, stability studies of protected alcohols, which assessed the retention of protecting groups under various deprotection conditions, demonstrated that HSDMS ether exhibits stability comparable to TMS ether, while HSDIS ether closely resembles TIPS ether in stability, which suggests that HSDIS provides greater resistance to degradation.

**Scheme 17:**
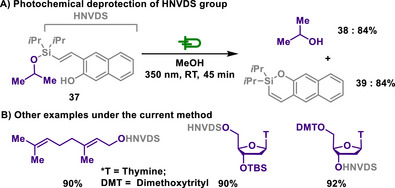
Photochemical deprotection of HNVDS group.

Although styrylsilyl ether protection demonstrated remarkable robustness and versatility in synthetic applications, it had a significant drawback due to its requirement for intense UV light at 254 nm. To address this limitation, Pirrung and Lee devised (2‐hydroxy‐3‐naphthyl)vinyl)diisopropylsilyl (HNVDS) group.^[^ ^126^ ^]^ By simply replacing phenol with 2‐naphthol, milder photochemical reaction conditions were accomplished using 350 nm light source, thereby broadening its practical utility in synthetic chemistry (Scheme 17).

**Scheme 18:**
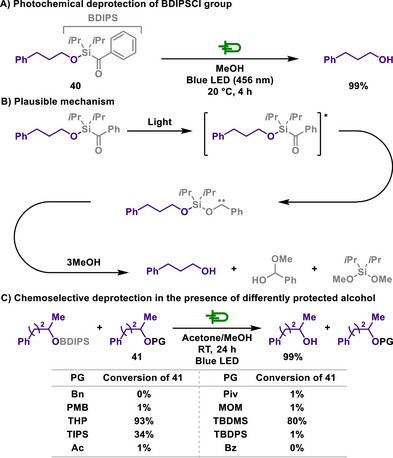
Photochemical deprotection of BDIPSCl group.

Building on these advancements, the Studer group introduced an alternative photoremovable silyl protecting group, benzoyldiisopropylchlorosilane (BDIPSCl), which operates under significantly milder light conditions (Scheme 18).^[^ ^127^ ^]^ Unlike the previous styrylsilyl ether systems requiring intense UV irradiation, BDIPSCl enables deprotection under visible light region of 456 nm, broadening its applicability in synthetic chemistry. Moreover, while styrylsilyl ether deprotection relies on *cis‐trans* isomerization or photosolvolysis, BDIPSCl undergoes a distinct photochemical cleavage mechanism involving aryl silane rearrangement to siloxy carbenes. This mechanistic divergence highlights the expanding versatility of photoremovable silyl protecting groups, providing chemists with a diverse toolbox for selective alcohol deprotection. For instance, the effectiveness of the reaction manifold was successfully demonstrated in the deprotection of primary, secondary, and tertiary alcohols, while further exploration revealed that the method exhibits excellent orthogonality with other commonly used alcohol protecting groups, ensuring its broad applicability in complex synthetic sequences.

#### 2.2.6 Allyl Deprotection

Since allylation is also a common protection method for alcohols, various methods exist for the C─O bond cleavage of allyl ethers for the purpose of their deprotection.^[^ ^128^ ^]^ The deprotection manifolds typically include basic or acidic conditions, or hydrogenation. However, it is noteworthy that the reaction pathway with a strong base, such as KO*t*Bu, undergoes double bond isomerization, forming enol ether as reaction intermediate.

**Scheme 19:**
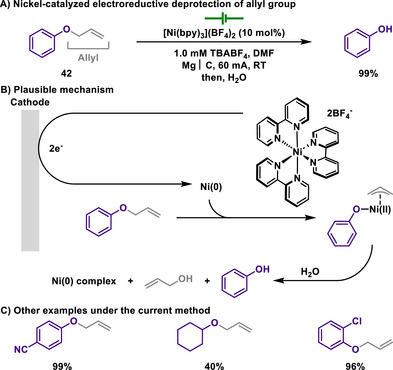
Nickel‐catalyzed electroreductive deprotection of allyl group.

**Scheme 20:**
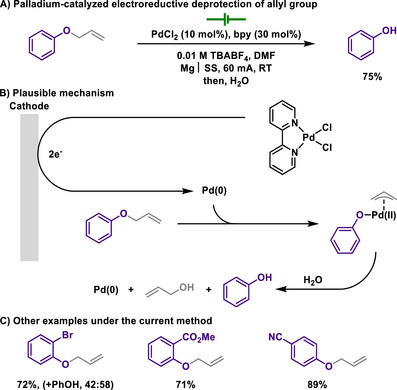
Palladium‐catalyzed electroreductive deprotection of allyl group.

In connection with this, Duñach reported that electroreductive nickel catalysis effectively enabled the deprotection of allyl groups under mild conditions at room temperature, in which sensitive functional group‐containing substrates were fully tolerated, for example, esters, halides, or nitriles (Scheme 19).^[^ ^129^ ^]^ The authors proposed that a well‐defined nickel(II) complex underwent electrochemical reduction, followed by oxidative addition of the C(allyl)─O bond, producing *π*‐allyl‐Ni(II) intermediates as key catalytic species. The existence of *π*‐allyl‐Ni(II) intermediates was further supported, as the electrolysis of allyl 2‐chlorophenyl ether in the presence of pivaldehyde led to the formation of *tert*‐butyl homoallyl alcohol. Through this mechanism, the need for strong bases typically required in conventional allyl group deprotection was eliminated, thereby achieving high functional group tolerance. Later, the same group showed the allyl deprotection by using palladium catalysis through a similar mechanistic pathway (Scheme 20).^[^ ^130^ ^]^

#### 2.2.7 Tosyl Deprotection

Tosyl group is a prominent protecting group that is utilized in alcohols and amines due to its high stability. Tosylation generally requires basic conditions to facilitate the deprotonation of the functional groups, thus forming tosyl‐protected alcohols and amines. On the other hand, traditional methods for the deprotection of the tosyl group require forcing conditions, typically involving strong acids or strong bases at elevated temperatures, due to the high stability of the resulting tosylated alcohols or amines. Alternatively, reduction‐based approaches have also been explored for tosyl deprotection. This often involves the use of various chemical reductants, such as alkali or alkaline‐earth metals and samarium(II) iodide. However, due to the consumptive and environmentally demanding nature of these reducing agents, alternative strategies employing electrochemical or photochemical methods for the removal of the tosyl protecting group have also been developed.

**Scheme 21:**
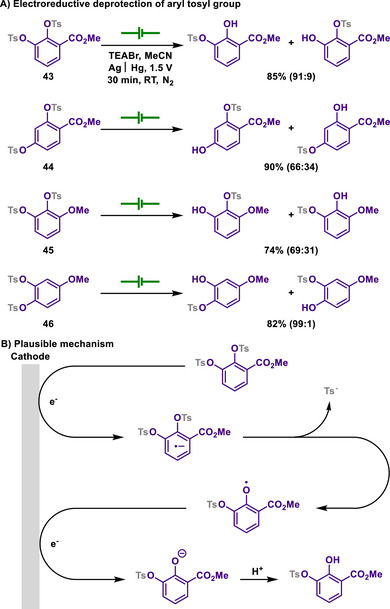
Electroreductive deprotection of aryl tosyl group.

In this regard, Rapoport group developed a straightforward electrochemical reduction method for the detosylation with mercury as an electrode (Scheme 21).^[^ ^131^ ^]^ This reaction manifold provides site‐selective cleavage from bis‐tosyloxy compounds.

Interestingly, the position of the deprotected tosyl group in the resulting products was influenced by the nature of substituents of arenes. For example, electron‐withdrawing substitution promoted the cleavage of tosylates at the *ortho*‐ and *para*‐positions, while electron‐donating substituents tended to favor the cleavage at the *meta*‐position. The author proposed that this tendency is attributed to the stability of the phenoxide ion formed as an intermediate. In the case of hydroxyanisole, which can form a *p*‐benzoquinone‐type intermediate, the expected compound from detosylation is difficult to synthesize. During the course of electrolysis, two sequential and irreversible reduction waves with a potential difference of approximately 300–500 mV were observed. This phenomenon is attributed to the phenoxide ion formed after the cleavage of the first tosyl group, which renders the reduction of the second tosyl group more difficult.

**Scheme 22:**
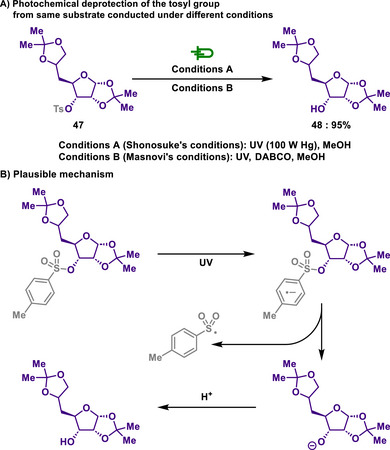
Photochemical deprotection of the tosyl group from the same substrate conducted under different conditions.

In 1968, Shonosuke introduced a novel photochemical method for the detosylation of carbohydrates (Conditions A, Scheme 22).^[^ ^132^ ^]^ In their experiment, a methanol solution of the sugar tosylate was treated with an equimolar amount of methoxide and irradiated for five hours under a nitrogen atmosphere using a 100 W high‐pressure mercury lamp. The results of this study demonstrated that detosylation could be successfully achieved through a photochemical reaction without any side reactions. Later, Masnovi successfully accomplished the photochemical detosylation of protected carbohydrates (Conditions B, Scheme 22), and through further investigation, elucidated the underlying mechanism of the process.^[^ ^133^ ^]^ The authors proposed that the electron‐donating ability of the amine facilitated the deprotection process, while its nucleophilicity had no discernible effect. Initially, proposed mechanisms involved homolytic cleavage of the S─O bond to form an alkoxy radical. However, analysis of the absorption spectrum from flash photolysis of tosylate revealed a 320 nm peak attributable to a tosyl radical, indicating the reaction proceeds via the formation of an alkoxide rather than an alkoxy radical. Particularly, DABCO interacts with the photoexcited state of the tosyl‐protected compound, donating an electron, which subsequently leads to the formation of a DABCO radical cation and a *p*‐tolylsulfonyl radical. Furthermore, experiments using electron‐donating photosensitizers did not enhance the electron transfer of carbohydrate tosylate, suggesting this could be attributed to both the thermodynamic and stereochemical effects of carbohydrates.

#### 2.2.8 PMP Deprotection

**Scheme 23:**
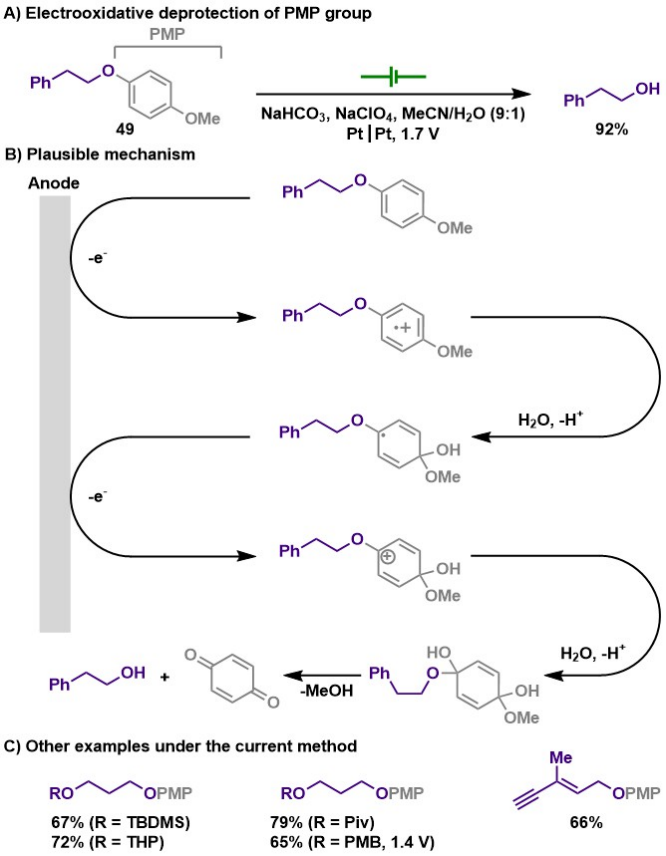
Electrooxidative deprotection of PMP group.

The *para*‐methoxyphenyl (PMP) ether possesses unique features as a protecting group for alcohols, offering stability under strong acidic and basic conditions while being selectively cleaved under oxidative conditions.^[^ ^134^, ^135^ ^]^ In this sense, Ardisson and Royer introduced a method for the deprotection of PMP ethers via anodic oxidation.^[^ ^136^ ^]^ This electrochemical method provides a higher efficiency and reproducibility compared to a traditional way of using ceric ammonium nitrate (CAN) for the same chemical transformation. Moreover, constant potential electrochemical oxidation allows a wide range of functional group tolerances that are usually sensitive under nonneutral reaction conditions, thereby highlighting the advantage of electrochemical PMP deprotection method. Based on the CAN‐mediated PMP cleavage reaction, the authors also proposed an electrochemical stepwise oxidation process, leading to the formation of a quinone and the projected alcohols (Scheme 23).

## 3 Amines

An amine is an organic compound derived from ammonia in which one or more hydrogen atoms are replaced by carbon‐based alkyl or aryl groups. Starting from the foundational Gabriel synthesis,^[^ ^137^ ^]^ taught in organic chemistry textbooks, numerous amine synthesis methods have been developed. These amine scaffolds play essential roles in ranging from simple organic molecules to pharmaceuticals and materials. Particularly in organic synthesis, amines often act as nucleophilic agents, which frequently necessitates the use of protective strategies to control undesired side reactions during specific transformations. Consequently, the development of efficient protecting groups and selective deprotection methods has become a crucial aspect in the construction of synthetic strategies, enabling the precise manipulation of amines in complex molecular architectures.^[^ ^138^ ^]^

### 3.1 Protection

#### 3.1.1 Tosyl Protection

As discussed in section 2.2.7, amines can also be protected by a tosyl group. Sulfonamides are generally prepared by reacting amines with sulfonyl chlorides under basic conditions. Unfortunately, sulfonyl chlorides possess toxicity and instability, and their preparation typically requires highly reactive chlorinating agents, such as SOCl_2_ or PCl_5_, which can be often hazardous to handle.

**Scheme 24:**
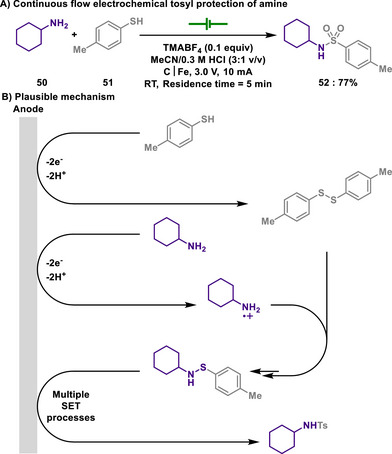
Continuous flow electrochemical tosyl protection of amine.

In sharp contrast, the Noël group devised an electrochemical approach for the tosyl protection of amines with easily accessible *p*‐thiocresol, thus alleviating the need for corrosive and fuming chemical reagents (Scheme 24).^[^ ^139^ ^]^ In their experimental mechanistic studies, the absence of sulfonamide formation upon the addition of radical scavengers such as TEMPO and 1,1‐diphenylethylene suggests that the aminium radical cation is a key intermediate in the reaction. Salient feature of this development is that a microflow reactor was employed, enabling the efficient and scalable tosyl protection of amines. The reaction began with the oxidation of thiols **51** and amines **50** to form an S─N bond and the subsequent oxidation produces tosyl‐protected amines. Thus, various amines, ranging from simple ammonia to complex amino acids, were successfully protected in the form of sulfonamides, indicating a broader spectrum of reactivity.

#### 3.1.2 Amide Protection

Similar to sulfonamides, a simple amide is also a viable form to protect an amine functional group. In general, the amide protection can be achieved using corresponding anhydrides or acyl halides under basic conditions, and deprotection is readily achieved under acidic conditions. Trifluoroacetyl group also gives a similar protecting ability, but an electron‐withdrawing property enables a deprotection process rather easier.

**Scheme 25:**
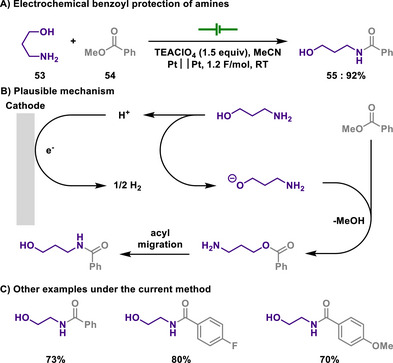
Electrochemical benzoyl protection of amines.

In 1990, the Nakajima group successfully demonstrated the benzoylation of amines by employing an electrogenerated base under exceedingly mild conditions at room temperature (Scheme 25).^[^ ^140^ ^]^ Interestingly, this reaction pathway required the presence of an alcohol moiety in the substrate in the form of amino alcohols. Therefore, electrochemical transesterification followed by *O*,*N*‐acyl migration led to the desired amidated product, which proved that amino alcohols with protected hydroxyl groups did not yield the amidated product. However, in the case of *ortho*‐ and *meta*‐aminobenzyl alcohols, the direct attachment of the amine to the benzene ring reduces its nucleophilicity, preventing *O*,*N*‐acyl migration, thereby resulting in the formation of benzoic acid as the final product.

**Scheme 26:**
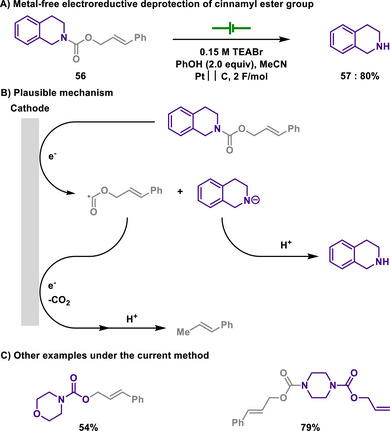
Metal‐free electroreductive deprotection of cinnamyl ester group.

### 3.2 Deprotection

#### 3.2.1 Carbamate Deprotection

Boc, *tert*‐butoxycarbonyl, is a common protective group for amine functional groups.^[^ ^141^ ^]^ The protection process is readily achieved by reacting di‐*tert*‐butyl dicarbonate with amines in the presence of an appropriate base, generating concomitant carbon dioxide. One of the key advantages of introducing the Boc group for amine protection is its ease of removal, which can be efficiently achieved under mild acidic conditions, such as treatment with trifluoroacetic acid (TFA) or acyl chloride. In the realm of protection of amines as carbamates, fluorenylmethoxycarbonyl (Fmoc) group is also a viable candidate, that can be cleaved under basic conditions in which the deprotonation of acidic C─H bond leads to decarboxylative removal of Fmoc group.

As an extension of this discussion, cinnamyloxycarbonyl (COC) moiety could also be utilized for the purpose of amine protection. The cinnamyl group is utilized for protecting various functional groups such as alcohols, carboxylic acids, and amines. Its typical deprotection method involves hydrogenation reactions using palladium catalysts. Although electrochemical deprotection of cinnamyl groups with palladium catalysts has been reported, Freeman group demonstrated a method for selective electrochemical deprotection of the COC group without metal catalysts (Scheme 26).^[^ ^142^ ^]^ Notably, the installation of COC group enabled to discriminate a similar protecting moiety, allyloxycarbonyl, during the course of voltage‐controlled electroreduction, thus achieving a selective deprotection. Unfortunately, cinnamyl carbonate and carbamate in the same compound were not discriminated, indicating that C─O or C─N bond cleavage of the reaction is solely controlled by the nature of cinnamyl protecting group. Moreover, the deprotection of *O*‐cinnamyl protection occurs more readily than that of *N*‐cinnamyl protection. This is because the cinnamyl protection attached to oxygen forms an alkoxide, which has lower basicity compared to an amide, rendering its removal relatively easier.

**Scheme 27:**
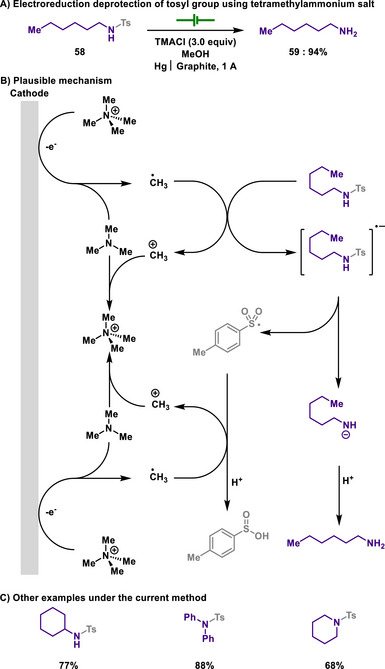
Electroreduction deprotection of tosyl group using tetramethylammonium salt.

#### 3.2.2 Tosyl Deprotection

In 1965, Horner and Neumann disclosed an electrochemical process in which they proposed that tetramethylammonium salt serves as a redox mediator for the selective reduction of various organic compounds (Scheme 27).^[^ ^143^ ^]^ A key focus of their work is the detosylation reaction facilitated by the ammonium salt, which provides a mild and selective means of removing tosyl protecting groups.

During the electrolysis at a mercury cathode, TMA undergoes electron transfer, leading to its decomposition into trimethylamine and a highly reactive methyl radical. They proposed that the methyl radical reduces tosyl‐protected amines, thereby enabling S─N bond cleavage process. This reaction manifold was further extended to peptide chemistry, in which minimal damage to the peptide backbone was observed, ensuring the preservation of the stereochemical integrity of amino acids during the selective cleavage of tosyl protecting groups. Notably, similar studies by the Jeminet group demonstrated the removal of arylsulfonyl groups from sulfonamides through a two‐electron reduction pathway, highlighting a complementary approach to sulfonyl group deprotection.^[^ ^144^, ^145^ ^]^

**Scheme 28:**
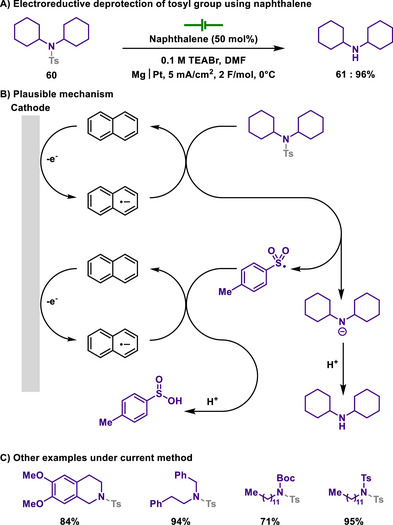
Electroreductive deprotection of tosyl group using naphthalene.

In 2010, Senboku group replaced the ammonium salt with naphthalene and the mercury electrode with a sacrificial magnesium anode, thus allowing detosylation reaction in an undivided cell under neutral and mild conditions (Scheme 28).^[^ ^146^ ^]^ In this reaction, naphthalene was used as a redox mediator and an active reductant, enabling that tosyl‐protected amines are efficiently reduced. Therefore, N─S bond of tosylamide is concomitantly cleaved, eventually forming deprotected amines and sulfinic acid. Indeed, this straightforward and efficient reaction has the potential to replace traditional detosylation methods. Although conventional methods for the mono‐detosylation of *N*‐alkyl‐di‐tosylamides typically require harsh reducing conditions, the described reaction facilitates selective mono‐detosylation under milder conditions, providing a new synthetic strategy toward *N*,*N*‐dialkylamines.

**Scheme 29:**
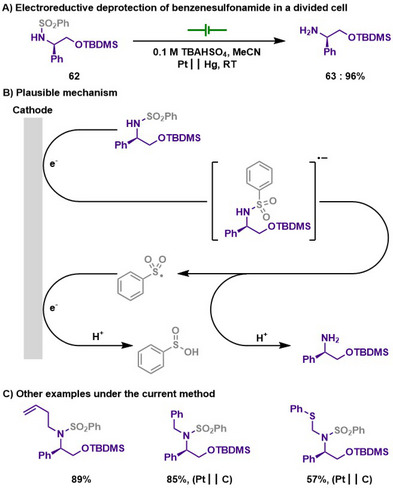
Electroreductive deprotection of benzenesulfonamide in a divided cell.

Later, Grognec and Quintard also showed electroreductive deprotection of the benzenesulfonyl group in a divided cell under mild conditions (Scheme 29).^[^ ^147^ ^]^ Similar to the previous studies, single electron reduction induced the S─N bond scission, thereby achieving deprotection of the arylsulfonyl group. As discussed in section 2.2.7, SmI_2_ is a common choice for the deprotection of arylsulfonyl groups, but the author demonstrated that chemical deprotection conditions are less likely operative for the removal of benzenesulfonyl groups. Therefore, the authors were able to propose an alternative approach that addresses the limitations of conventional deprotection methods while achieving sustainable chemistry.

## 4 Carboxylic Acids

Carboxylic acids are organic compounds characterized by the presence of a carboxyl group, exhibiting a wide range of organic molecules.^[^ ^148^ ^]^ These compounds are highly soluble in water and can engage in intermolecular hydrogen bonding, facilitating their interaction in various environments. In synthetic chemistry, carboxylic acids function as attractive starting materials and intermediates, contributing to the production of various compounds, ranging from pharmaceuticals to commodities. Recently, carboxylic acid‐containing molecules, such as lactic acid, have gained significant attention in biofuel production. Therefore, more sustainable protection and deprotection of carboxylic acids can help enhance their efficient utilization across various industries.

### 4.1 Deprotection

#### 4.1.1 Ester Deprotection

The protection of carboxylic acids can be easily achieved through esterification with alcohols or amidation with amines. These protected acids can be deprotected through simple hydrolysis reactions; however, they typically require the use of strong bases or strong acids.^[^ ^149^ ^]^

**Scheme 30:**
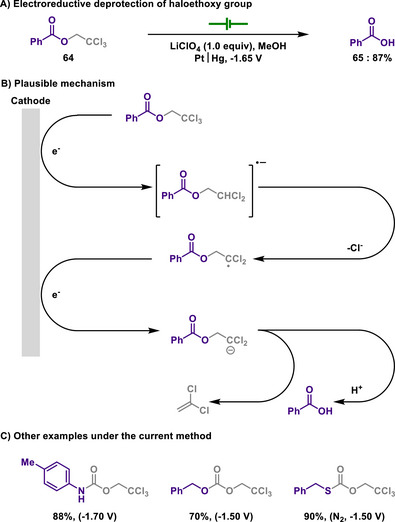
Electroreductive deprotection of haloethoxy group.

In sharp contrast, Semmelhack showed a method for the selective removal of 2‐haloethoxy protecting groups using controlled potential electrolysis (Scheme 30).^[^ ^150^ ^]^ In this study, electrochemical deprotection was successfully achieved for carboxylic acids protected with trichloroethoxy moiety. They proposed that electroreduction enables C─O bond cleavage, thus resuming acids and concomitantly generating 1,1‐dichloroethylene. Since this deprotection manifold highly relies on the type of halogen, electrolysis under precisely controlled potential is necessary for gaining chemoselectivity. Interestingly, the developed deprotection method was further extended to other functional groups, including amines, alcohols, and thiols, which were protected with 2,2,2‐trichloroethoxycarbonyl (Troc) protecting group.

#### 4.1.2 Benzyl Deprotection

**Scheme 31:**
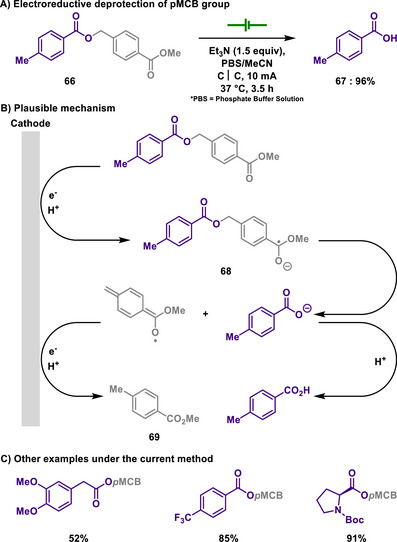
Electroreductive deprotection of *p*MCB group.

In sharp contrast, the Xiang group devised an electro‐removable protecting group, *p*‐methoxycarbonylbenzyl (*p*MCB), for acid functional groups, which could be further extended to other functional groups such as phosphoric acids and alcohols (Scheme 31).^[^ ^151^ ^]^ Notably, this study was conducted at a physiological temperature of 37 °C and a pH of 7.4–7.5 to ensure its applicability in biological research. The cathodic reduction of esters leads to C─O bond cleavage, forming acids and the corresponding protecting group, *p*MCB. This process was further examined through a reaction with the radical scavenger TEMPO. Particularly, the author proposed that two distinct reduction peaks observed in cyclic voltammogram studies are attributed to the formation of **68** and **69**. This reaction condition selectively deprotects only the *p*MCB group without affecting other common protecting groups such as Boc, CBz, Ac, and Bn, thereby highlighting its orthogonality and utility in synthetic strategies involving multiple protection‐deprotection sequences.

#### 4.1.3 Photolabile Group Deprotection

**Scheme 32:**
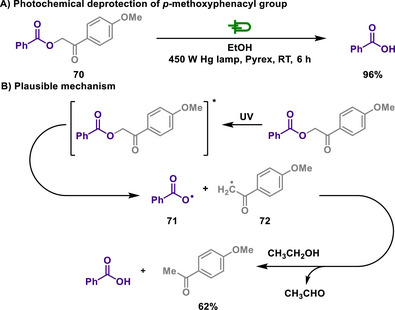
Photochemical deprotection of *p*‐methoxyphenacyl group.

Indeed, PPGs, discussed in section 2.2.1., offer a significant advantage due to their ability to be removed under mild reaction conditions. Concerning this, Sheehan investigated the photolysis of photosensitive phenacyl groups (Scheme 32).^[^ ^152^ ^]^ The authors observed that there were no observable reactions typical of the general photolysis of *p*‐methoxyphenacyl ester **70**, and the starting material was recovered. However, upon conducting the photoreaction with a Pyrex filter, which filters shorter wavelengths effectively, it was observed that the ester underwent decomposition to yield benzoic acid and acetophenone compounds. The reaction undergoes the formation of two radicals, **71** and **72,** under UV irradiation, which are reduced to benzoic acid and a phenacyl compound, respectively. In this process, ethanol plays two roles: a hydrogen donor and reductant. Unsubstituted phenacyl esters exhibited lower photolysis efficiency compared to *p*‐methoxyphenacyl esters. The authors suggested that the methoxy substitution induces a bathochromic shift, reducing the required radiation energy, and that the radical intermediate of substituted phenacyl esters is further stabilized by the electron‐donating effect of the methoxy group.

**Scheme 33:**
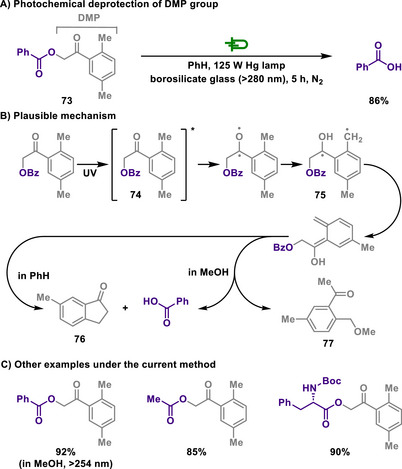
Photochemical deprotection of DMP group.

Klán introduced the 2,5‐dimethylphenacyl (DMP) group as another photoremovable protecting group for carboxylic acids (Scheme 33).^[^ ^153^ ^]^ Photolysis of DMP esters could be carried out in methanol or benzene as a reaction medium, but the quantum yield in methanol was approximately 1.5 times lower than that in benzene. Further studies have expanded the applicability of the DMP group to phosphoric and sulfonic acid esters, suggesting its broad potential for use not only in organic chemistry but also in biochemical contexts.^[^ ^154^ ^]^

The reaction is initiated when the DMP ester was excited to triplet state **74** upon irradiation. A subsequent intramolecular hydrogen abstraction occurred from an adjacent methyl group, leading to the radical intermediate **75**. After this, **75** releases the carboxylic acid with rearrangement via an enol intermediate, ultimately yielding 6‐methyl‐1‐indanone **76**. The formation of **76** as a photolysis product is consistent with the earlier findings reported by Bergmark.^[^ ^155^ ^]^ In contrast, when the reaction is performed in the nucleophilic methanol, photosolvolysis from the triplet excited state preferentially occurs, resulting in the formation of a methoxy‐substituted byproduct **77**. This study demonstrates that the DMP group serves as an excellent protecting group in organic synthesis, offering high deprotection yields, broad wavelength applicability, and superior photochemical efficiency.

**Scheme 34:**
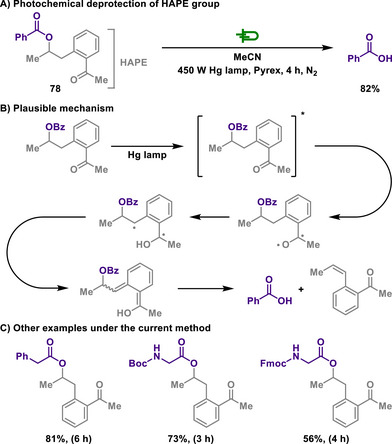
Photochemical deprotection of HAPE group.

Banerjee group introduced 1‐[2‐(2‐hydroxyalkyl)phenyl] ethenone (HAPE) as a photoremovable protecting group for carboxylic acids and investigated its application in carboxylic acid protection and photochemical deprotection reactions (Scheme 34).^[^ ^156^ ^]^ The photochemical deprotection mechanism of the HAPE protecting group follows a process analogous to the Norrish type II reaction, wherein carboxylic acid is released via *γ*‐hydrogen abstraction.

Upon irradiation of UV, HAPE esters transition to an excited state, in which gamma‐hydrogen abstraction occurs, leading to the formation of the enol intermediate.^[^ ^157^ ^]^ This enol can exist in either the *E* or *Z* isomeric form. Notably, Klán's study showed that the E‐enol form of 2′‐methylphenacyl ester tautomerizes much more slowly than the Z‐form.^[^ ^153^ ^]^ Due to significant steric hindrance and the possibility of rearrangement, *Z*‐enol form either undergoes deprotection inefficiently or does not undergo deprotection at all. Consequently, only the *E*‐enol form participates effectively in the carboxylic acid release reaction. Furthermore, the deprotection reaction was suppressed in the presence of oxygen, whereas photodegradation proceeded exclusively under a nitrogen atmosphere. These observations confirmed that the triplet excited state is quenched by oxygen, inhibiting the deprotection reaction, and thus indicating that the triplet state is the predominant reaction pathway.

## 5 Amides

Amide is one of the derivatives of carboxylic acids in which the hydroxyl group of the carboxyl group is replaced by an amine group. Amides play a critical role across various fields, including chemistry, medicine, and materials science. Notably, they hold particular significance in biochemical processes, as the peptide bonds that link amino acids in proteins are a specific type of amide bond. Due to their involvement in the structure and function of proteins, amides are essential to biomolecular research.

### 5.1 Deprotection

#### 5.1.1 Benzyl Deprotection

Similar to the protection and deprotection processes for amine functional groups, discussed in section 3, the amine portion of an amide can also be protected using groups such as benzyl, Boc, or Fmoc, and accordingly deprotected through sustainable approaches.

**Scheme 35:**
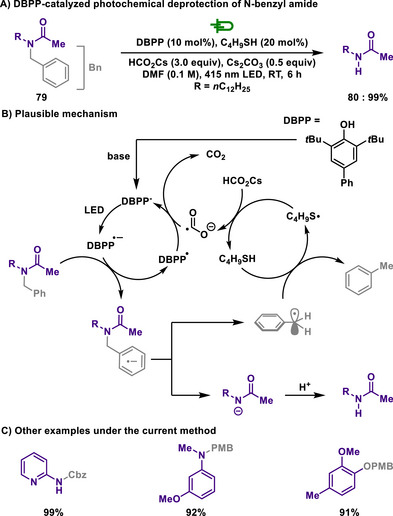
DBPP‐catalyzed photochemical deprotection of *N*‐benzyl amide.

On this matter, Xia developed a photochemical method (Scheme 35)^[^ ^158^ ^]^ for the removal of benzyl group from amides. The excited state of a photocatalyst, 2,6‐di‐*tert*‐butyl‐4‐phenylphenolate (DBPP), has a strong reduction potential (−3.16 V vs. SCE), thereby generating phenyl radical anions and leading to a subsequent mesolytic C─N bond cleavage. Thus, formed benzyl radical obtained hydrogen from a thiol catalyst that is transformed to thiyl radical. In this process, hydrogen atom transfer followed by a SET of cesium formate enables the catalytic system to operate. Interestingly, not only were benzyl‐protected amines effectively debenzylated, but so were benzyl‐protected alcohols, carboxylic acids, and esters. Furthermore, various benzyl‐derived groups such as Cbz or PMB were easily applied, making them applicable in diverse industrial fields. The reaction does not proceed with *N*‐benzyl aliphatic amines due to their lower reduction potential (−3.2 V vs. SCE) compared to DBPP, preventing electron transfer. However, converting them into *N*‐benzyl ammonium salts facilitates electron transfer, thus enabling reaction progress.

#### 5.1.2 Tosyl Deprotection

Tosyl‐protected amides can also undergo photochemical cleavage of the N─S bond, enabling selective and efficient deprotection under mild conditions.

**Scheme 36:**
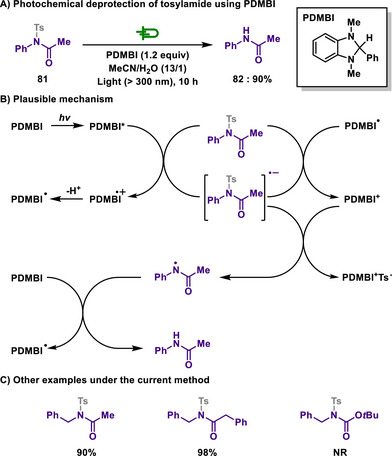
Photochemical deprotection of tosylamide using PDMBI.

Liu and Yu utilized 2‐phenyl‐*N*,*N*'‐dimethylbenzimidazoline (PDMBI), which has been extensively investigated as a biomimetic reducing agent, as both an electron and hydrogen donor for tosyl deprotection (Scheme 36).^[^ ^159^ ^]^ The reaction is initiated by a single‐electron transfer from photoexcited PDMBI. The resulting unstable PDMBI radical cation, generated by the transfer of an unpaired electron from PDMBI, undergoes mesolytic cleavage, leading to deprotonation and the formation of a tosylamide radical anion. This intermediate then undergoes anionic mesolysis, resulting in detosylation and the formation of a neutral radical. Subsequently, it accepts a hydrogen atom from PDMBI, yielding the final product. Under these reaction conditions, *N*‐tosyl‐protected free amines remained unaffected, whereas *N*‐tosyl‐protected amides underwent deprotection. This selective detosylation was further extended to the differentiation of amino and amido groups when both are tosylated.

## 6 Diols/Amino Alcohols

Acetals are formed by the reaction of aldehydes or ketones with diols or amino alcohols and are commonly used in organic synthesis as protecting groups.^[^ ^160^ ^]^ Their high stability under basic conditions prevents aldehydes and ketones from undergoing undesired reactions during synthetic processes. Once the desired transformations are complete, acetals can be hydrolyzed under acidic conditions to regenerate the original aldehyde or ketone.

### 6.1 Protection

#### 6.1.1 Acetal Protection

**Scheme 37:**
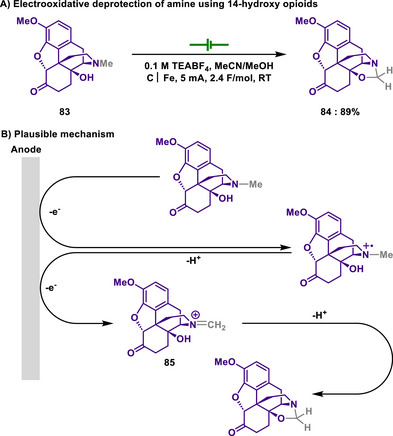
Electrooxidative deprotection of amine using 14‐hydroxy opioids.

In 2020, Kappe and Cantillo reported a catalyst‐ and reagent‐free electrochemical method for *N*‐demethylation of 14‐hydroxy opioids, a key step in synthesizing opioid antagonists (Scheme 37).^[^ ^161^ ^]^ Compared to previous nonelectrochemical methods, this approach avoids toxic, corrosive, and potentially carcinogenic reagents. Similar to the Shono oxidation, the *N*‐methyl group of oxycodone is anodically oxidized to generate iminium ion **85**, which is rapidly intercepted by the C14‐hydroxyl group, forming the oxazolidine intermediate **84**. Cyclic voltammetry studies confirmed the selective oxidation of the tertiary amine, minimizing undesired side reactions such as aromatic ring oxidation and dimer formation. This methodology was also successfully scaled up using a continuous flow electrolysis cell, demonstrating high efficiency and reproducibility. Under batch conditions, electrochemical oxidation of *N*‐methyl tertiary amines often results in the formation of destabilized iminium intermediates, leading to undesired dimerization. In contrast, employing a flow electrolysis setup allows rapid stabilization of these intermediates, effectively suppressing dimerization. Despite its advantages, industrial application is limited by dimer formation necessitating chromatographic separation, the hazardous and nonrecoverable Et_4_NBF_4_ electrolyte, and health concerns associated with the MeCN/MeOH solvent system. Additionally, the process yields noroxycodone, requiring a subsequent *O*‐demethylation step for naloxone and naltrexone synthesis. The authors' follow‐up study revealed that these factors were eliminated, allowing the reaction to be redefined by optimizing the electrolyte and solvent system. Using KOAc as a supporting electrolyte and ethanol as a solvent minimized dimer formation, prevented overoxidation, thus significantly enhancing yield, and eliminated the reliance on large‐molecular‐weight salts like Et_4_NBF_4_. Additionally, integrating the electrochemical step with HBr‐mediated *O*‐demethylation streamlined the process, removing the need for intermediate isolation and hazardous reagents such as BBr_3_ or L‐selectride.

### 6.2 Deprotection

#### 6.2.1 Acetal Deprotection

**Scheme 38:**
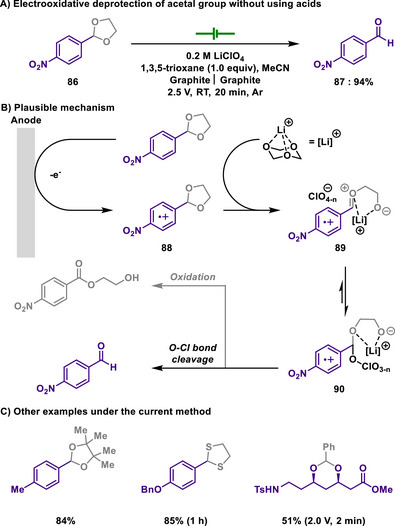
Electrooxidative deprotection of acetal group without using acids.

Generally, acids are required for the deprotection of acetals. However, the Yamada and Sajiki groups have developed an electrochemical deprotection of acetals, in which the reaction proceeds rapidly under neutral conditions without the use of acid (Scheme 38).^[^ ^162^ ^]^ In this deprotection manifold, lithium perchlorate acts as both an electrolyte and a source of oxygen, while 1,3,5‐trioxane functions as an activator for lithium ions, thereby enhancing the efficacy of the deprotection reaction. This method is initiated by anodic oxidation, in which one‐electron oxidation generates the radical cation intermediate **88** that subsequently binds with activated lithium ions to form the oxocarbenium intermediate **89**. Nucleophilic species generated from perchlorate further react with the oxocarbenium intermediate to form intermediate **90**, which undergoes cleavage of the Cl─O bond to yield aldehyde **87**. Intermediate **90** may also undergo further oxidation to form an ester. The addition of water or oxygen gas promotes unnecessary oxidation, leading to an increased yield of byproducts. Notably, this versatile approach was also applicable to pinacol and dithiol acetals, and when applied to compounds containing both acetal and ketal groups, it successfully removed both. Importantly, the utility of this method was demonstrated in pharmaceutical synthesis. When applied to a precursor of atorvastatin, the electrochemical deprotection proceeded without affecting the steroidal protecting group and preserved the compound's stereochemical integrity, underscoring its compatibility with structurally sensitive and functionally dense molecules.

**Scheme 39:**
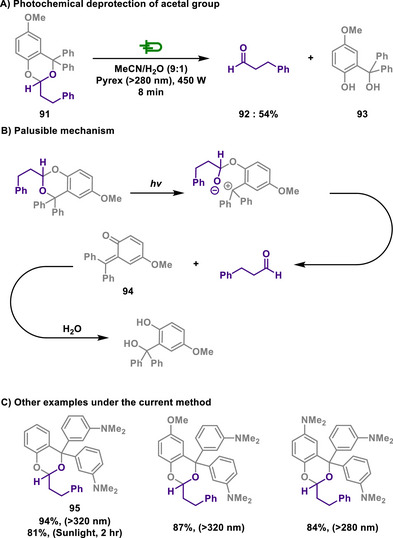
Photochemical deprotection of acetal group.

Building on this advancement in electrochemical acetal cleavage, photochemical strategies have also emerged as complementary, mild, and selective approaches (Scheme 39).^[^ ^163^ ^]^ The reaction proceeded through photolytic cleavage of the benzylic C─O bond under light. The byproduct **94** was recovered as salicylic alcohol **93** through its reaction with water. The introduction of an electron‐donating group at the *meta*‐position of the salicylic alcohol skeleton facilitated the cleavage of benzylic C─O bonds. Additionally, the 3‐(dimethylamino)phenyl moiety at the *α*‐position influenced the efficiency of photochemical deprotection. Furthermore, PPGs were classified into three groups based on their UV absorption profiles, allowing sequential removal under UV irradiation at different wavelengths. These PPGs are structurally simple, chemically stable, and can be installed and removed with high efficiency without the use of inorganic materials. Moreover, it has been reported that substrate **95** achieved a high yield of aldehyde through deprotection under sunlight, with no significant improvement observed upon increasing sunlight exposure time, while prolonged irradiation led to the decomposition of the PPG reagents.

## 7 Miscellaneous

**Scheme 40:**
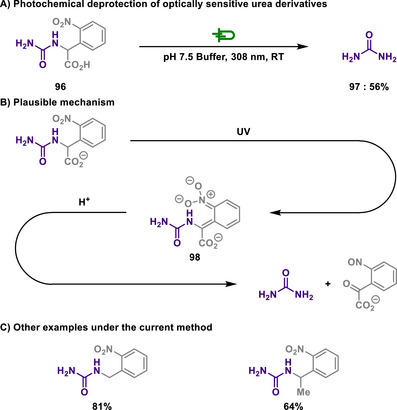
Photochemical deprotection of optically sensitive urea derivatives.

2‐Nitrobenzyl‐based protecting groups have become particularly prominent due to their versatility in shielding diverse functional groups such as carbamates, amines, carboxylates, phosphates, and amides.^[^ ^164^ ^]^ In this regard, Hess group extended this concept to the study of urea.^[^ ^165^ ^]^ They successfully synthesized several photolabile urea derivatives using an addition reaction between substituted 2‐nitrobenzylamines and isocyanates (Scheme 40). Upon UV irradiation, these urea derivatives undergo photochemical degradation of the 2‐nitrobenzyl protecting group, leading to the formation of a short‐lived intermediate **98**. This intermediate, characterized by an absorbance maximum between 420 and 440 nm, appears within sub‐microseconds and rapidly decomposes within 10–100 µs, releasing free urea and nitrogen‐containing photolysis byproducts. Importantly, these urea derivatives exhibit high photodegradation rates and excellent quantum yields. Additionally, the photolysis products do not affect the specific activity of urease, indicating compatibility with enzymatic assays. However, since the absorbance of photolysis products in the range of 300–400 nm exceeds that of the original caged compound, complete photolysis under continuous irradiation conditions could be limited.

**Scheme 41:**
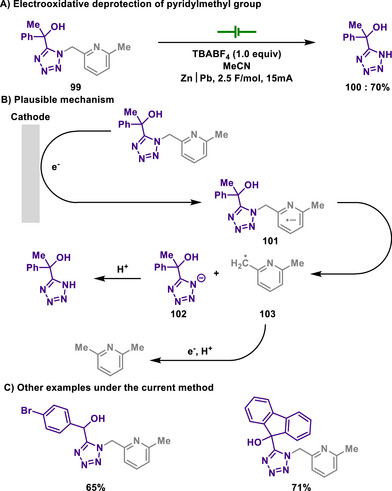
Electrooxidative deprotection of pyridylmethyl group.

Tetrazole derivatives play a significant role in a wide range of applications, including pharmaceuticals or high‐energy materials. Jirgensons employed a pyridylmethyl group as an electrochemically removable protecting group for tetrazole compounds (Scheme 41).^[^ ^166^ ^]^ During the electrolysis, pyridylmethyl‐protected tetrazole undergoes a cathodic reduction, generating radical anion species **101**. Subsequently, **101** decomposes into the tetrazole anion **102** and the pyridylmethyl radical **103**, which lead to desired deprotection process. Furthermore, the pyridylmethyl radical can undergo various secondary reactions, such as hydrogen atom abstraction, dimerization, oxidation, or further reduction, leading to the formation of multiple byproducts. In this reaction, maintaining low temperatures at −60 °C is essential, as raising the reaction temperature to ambient conditions resulted in rapid decomposition of the unstable tetrazole derivatives. Under these elevated‐temperature conditions, tetrazoles undergo degradation through a cyanamide intermediate, which subsequently cyclizes to form imidazopyridine derivatives, making the isolation of the desired tetrazole products difficult.

**Scheme 42:**
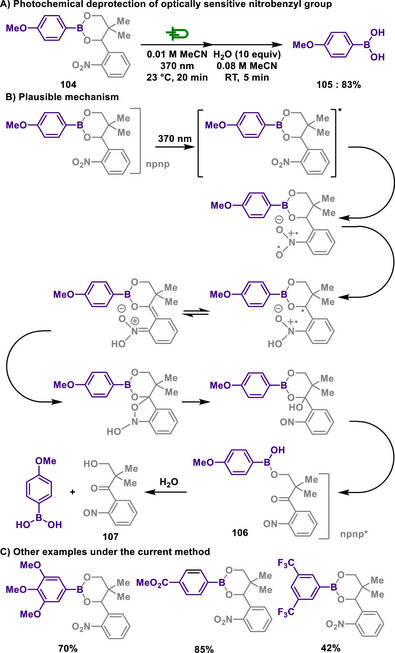
Photochemical deprotection of optically sensitive nitrobenzyl group.

Boronic and borinic acids require protection to regulate their Lewis acidity, enhance stability against hydrolysis and oxidation, and prevent side reactions during synthesis.^[^ ^167^, ^168^ ^]^ In this respect, Chabaud and Pucheault developed a novel PPG based on the nitrobenzyl moiety (Scheme 42).^[^ ^169^ ^]^ Notably, the protecting group utilizing 1‐(2‐nitrophenyl)neopentyl glycol (npnp) demonstrated efficient photochemical deprotection under mild conditions, exhibiting the most promising results in terms of synthetic efficiency, stability, and solubility. The npnp protecting group undergoes a Norrish II type‐rearrangement process upon exposure to light, leading to the formation of boronic acid hemiester intermediate **106**. The hemiester intermediate features a structure comprising boronic oxide and a nitrobenzyl derivative. Subsequently, the hemiester undergoes decomposition in the presence of moisture, resulting in the generation of boronic acid and the byproduct **107**. A major advantage of this approach is its simplicity, as it operates under mild reaction conditions without requiring a catalyst and utilizes simple solvents. Additionally, its versatility across various functional groups demonstrates high deprotection efficiency for both electron‐donating and electron‐withdrawing substituents.

## 8 Conclusion

In conclusion, electrochemical and photochemical approaches offer sustainable and efficient alternatives to conventional methods for the protection and deprotection of functional groups in organic synthesis. Their advantages, including mild conditions, improved selectivity, and broad functional group tolerance, address many of the limitations of traditional protocols. Additionally, the integration of continuous‐flow techniques with the modern synthetic tools enhances scalability and operational simplicity, offering clear benefits over batch processes.

Indeed, mechanistic insights gained from these methods allow for fine‐tuned reaction control, thus broadening their synthetic utility. Therefore, the continued development of these strategies together with the design of novel catalysts and innovative reaction systems will play a key role in advancing green and practical synthesis.

Ultimately, the long‐term objective should be the design of synthetic routes that minimize or eliminate the need for the introduction of protecting groups and their inevitable removal.^[^ ^170^, ^171^, ^172^, ^173^ ^]^ Progress in this direction will not only streamline synthesis but also reduce waste and environmental impact, aligning with the core principles of green chemistry.

## Author Contributions

I.C. and S.M.K. proposed the topic of the review. J.H.J., M.A., M.J., J.K., S.M.K., and I.C. wrote the manuscript.

## Conflict of Interest

The authors declare no conflict of interest.

## Data Availability

The data that support the findings of this study are available from the corresponding author upon reasonable request.
